# AATF and SMARCA2 are associated with thyroid volume in Hashimoto’s thyroiditis patients

**DOI:** 10.1038/s41598-020-58457-x

**Published:** 2020-02-04

**Authors:** Luka Brčić, Ana Barić, Benjamin Benzon, Marko Brekalo, Sanda Gračan, Dean Kaličanin, Veselin Škrabić, Tatijana Zemunik, Maja Barbalić, Ivana Novak, Valdi Pešutić Pisac, Ante Punda, Vesna Boraska Perica

**Affiliations:** 1 Department of Medical Biology, University of Split, School of Medicine, Split, Croatia; 2 Department of Nuclear Medicine, University Hospital of Split, Split, Croatia; 3 Department of Neurosciences, University Hospital of Split, Split, Croatia; 4 Department of Pediatrics, University Hospital of Split, Split, Croatia; 5 Department of Immunology and Medical Genetics, University of Split, School of Medicine, Split, Croatia; 6 Clinical Department of Pathology, Forensic Medicine and Cytology, University Hospital of Split, Split, Croatia

**Keywords:** Genome-wide association studies, Thyroid diseases

## Abstract

Thyroid volume of Hashimoto’s thyroiditis (HT) patients varies in size over the course of disease and it may reflect changes in biological function of thyroid gland. Patients with subclinical hypothyroidism predominantly have increased thyroid volume whereas patients with more pronounced hypothyroidism have smaller thyroid volumes. Suggested mechanism for thyroid atrophy is thyrocyte death due to apoptosis. We performed the first genome-wide association study (GWAS) of thyroid volume in two groups of HT patients, depending on levothyroxine (LT4) therapy, and then meta-analysed across. Study included 345 HT patients in total and 6 007 322 common autosomal genetic variants. Underlying hypothesis was that genetic components that are involved in regulation of thyroid volume display their effect in specific pathophysiologic conditions of thyroid gland of HT patients. We additionally performed immunohistochemical analysis using thyroid tissues and analysed differences in expression levels of identified proteins and apoptotic marker between HT patients and controls. We found genome-wide significant association of two loci, both involved in apoptosis, with thyroid volume of HT patients: rs7212416 inside apoptosis-antagonizing transcription factor *AATF* (P = 8.95 × 10^−9^) and rs10738556 near chromatin-remodeling *SMARCA2* (P = 2.83 × 10^−8^). In immunohistochemical analysis we observed that HT patients with homozygous *AATF* risk genotypes have decreased AATF expression (0.46-fold, P < 0.0001) and increased apoptosis (3.99-fold, P = 0.0001) in comparison to controls. HT patients with heterozygous *SMARCA2* genotypes have decreased SMARCA2 expression, albeit without reaching statistical significance (1.07-fold, P = 0.5876), and significantly increased apoptosis (4.11-fold, P < 0.0001). By two lines of evidence we show that two highly plausible genetic loci, *AATF* and *SMARCA2*, may be involved in determining the thyroid volume of HT patients. The results of our study significantly add to the current knowledge of disturbed biological mechanisms in thyroid gland of HT patients.

## Introduction

The main functional unit of thyroid gland is group of cells called follicle. Follicle consists of epithelial follicular cells, known as thyrocytes, which surround colloid filled with thyroid hormone precursor thyroglobulin. The size of follicles, number of thyrocytes and the size of colloid are all dependent on thyroid gland biological activity and can affect thyroid gland size^1,2^.

Thyroid volume can be enlarged in number of thyroid diseases^1^ whereas its size can also be decreased, as seen in progressed autoimmune hypothyroidism^3–5^. Moreover, Hashimoto’s thyroiditis (HT), also known as autoimmune thyroiditis, can be presented as goiter on one side of the spectrum or as atrophic thyroid gland on the other^4,6^. A study demonstrated that patients with autoimmune hypothyroidism have larger dispersion of thyroid volume size in comparison to controls indicating that some patients have increased and some decreased thyroid volumes, i.e. goiter and thyroid atrophy just represent the extremes in the distribution^3^. Further studies linked thyroid volume size with thyroid function, as it was found that patients with smallest thyroid volumes have more pronounced hypothyroidism^3,5^, whereas patients with subclinical hypothyroidism predominantly have increased thyroid volume^4^. It was also suggested that progression of autoimmune thyroiditis to overt hypothyroidism is accompanied by thyroid atrophy^4^. In other words, as thyroid volume changes over the course of disease^5^, it reflects the change in biological function of thyroid gland, as well as stroma changes, including lymphocitic infiltration, local edema, and vascularity. The underlying mechanisms of progressing atrophy are not well known, but the most suspected mechanism is thyrocyte death due to apoptosis^4,7^. In summary, thyroid mass is affected by the number of thyrocytes that are maintained through the balance between their proliferation and apoptosis^8^. However, predominant process in HT is increased apoptosis^9–12^. Although the knowledge on mechanisms and regulation of apoptosis in thyroid gland is limited, it is suggested that the most important apoptotic pathway includes the Fas death receptor^10^.

There are several other determinants of thyroid follicular cells volume and thyroid volume, including serum TSH and iodine^13,14^. In iodine sufficient areas, or in cases of iodine supplementation, thyroid volume of healthy individuals ranges within reference values^15,16^. Other factors that influence thyroid volume are age^17^, anthropometric features, such as body surface area (BSA)^18,19^, gender^20,21^, smoking^22^ and alcohol consumption^23^. Beside these, genetic factors are important regulators of thyroid size^13^. It has been estimated that genetic factors contribute to about 71% of the total variation in thyroid volume of healthy individuals^13^. Nevertheless, genetic determinants of healthy thyroid volume are not well understood and to date, only one genome-wide association study (GWAS) of thyroid volume in thyroid disease free individuals has been performed^24^. It identified four independent loci associated with thyroid volume located inside or near *CAPZB*, *FGF7* and *LOC440389*^24^. On the other hand, genetic factors that determine thyroid volume in thyroid diseases are completely unexplored.

This study, for the first time, analyzed genome-wide genetic variants underlying thyroid volume of HT patients. We hypothesize that in specific pathological conditions of thyroid gland, such as in HT, genetic components involved in the regulation of thyroid volume could actually be the same ones regulating the number of thyrocytes and thus, biological activity of thyroid gland. The main aim of our study was to identify genetic determinants underlying these processes and to verify findings by protein expression analysis in thyroid tissues that can further deepen the understanding of biology behind pathologic processes in HT.

## Methods

### Subjects

A total of 370 HT patients were involved in this study. Patients were recruited after complete examination by nuclear medicine specialist at the Outpatient clinic for thyroid disorders at the Department for Nuclear Medicine at the University Hospital Split (Croatia). All patients were of white European ancestry, adult and living in the southern Croatia, a region considered iodine sufficient since 2003^15^. Patients were diagnosed with HT on the basis of clinical examination and diffuse thyroid autoimmune disease established by characteristic thyroid ultrasound (US) imaging (unhomogenic thyroid tissue with diffusely reduced echo levels). Diagnosis was further complemented with biochemical measurements of thyroid hormones and antibodies (increased TSH, decreased T3, T4 and/or increased TPOAb and/or TgAb) according to ETA recommendations and guidelines for Management of Subclinical Hypothyroidism^25^. During recruitment period, from 2013 to 2016, we were sequentially recruiting newly diagnosed patients as they were referring to Outpatient clinic for thyroid disorders, mostly due to positive thyroid antibodies or abnormal TSH levels established during routine screenings, or because of suspected clinical manifestations of thyroid disorders. We additionally involved patients with previously diagnosed HT that came to physicians on follow-up. All HT patients that were recruited through our study are part of “Croatian biobank of patients with Hashimoto’s thyroiditis” (CROHT) that was established with principal aim of identifying genetic variants associated with HT^26^ and HT-related traits, such as thyroid antibodies^27^ and thyroid volume.

Dimensions of thyroid gland in HT patients were determined by thyroid ultrasonography performed using Medison Accuvix V10 (Samsung Medison Co., Ltd, Seoul 135–280, Korea) high frequency linear probe (8–12 MHz). Thyroid volume was calculated as a sum of volumes of both lobes of thyroid gland, where the volume of each lobe was calculated as length × width × depth × 0.479^28^. The body surface area (BSA) in m^2^ was estimated as W^0.425^ × H^0.725^ × 0.007184, where W is the weight in kg and H is the height in cm^29^. Thyroid hormones and antibodies levels in plasma of HT patients were determined by immunoassay reaction conducted in a fully automated instrument “Liaison” Biomedica Chemiluminescence Analyzer in the Laboratory of Biochemistry, University Hospital Split. Genomic DNA of HT patients was extracted from peripheral blood leukocytes, using Nucleon Genomic DNA Extraction Kit BACC3, according to the manufacturer’s instructions (GE Healthcare, Little Chalfont, Buckinghamshire, UK). DNA concentration was determined by the Nanodrop ND-1000 Spectrophotometer (ND-1000, Thermo Fisher Scientific, USA). The final concentration of DNA was uniformed across all samples and brought to 100 µg/µl prior genotyping.

Written informed consent was obtained from all study participants, and the study was approved by two separate Ethics Committees: the University of Split, School of Medicine (Classification no. 003-08/14-03/0001; Registry no. 2181-198-03-04-14-0028) and University Hospital Split (Classification no. 530-02/13-01/11; Registry no. 2181-147-01/06/J.B.-14-2). Both Ethics Committees declared that study is in accordance with the provisions of the Code of Ethics and the Helsinki Declaration.

### Clinical characteristics of HT patients

Descriptive statistics was used to summarize clinical characteristics of patients. None of continuous variables showed normal distribution (tested by Kolmogorov-Smirnov test), thus were presented as median (first quartile - third quartile), while nominal variables (gender) were presented as number of individuals (%) per category. To compare clinical characteristics between two groups of HT patients, depending on levothyroxine (LT4) therapy, Mann–Whitney-U test was used for continuous variables and χ^2^-test was used for nominal variables.

### Genotyping, quality control (QC) and imputation

All participants were genotyped using Illumina Infinium HumanCoreExome genotyping platform that contains 551 839 markers. We performed quality control (QC) of genotype data, using PLINK and R software, following the standard procedure.

In the sample QC, we excluded all samples with call rate less than 95% and all samples with heterozygosity rate deviating more than three standard deviations from the mean. We cross-checked reported sex with the sex inferred from the genotypes – no exclusions were made based on this criterion. We checked for ethnicity by performing multidimensional scaling analysis (MDS) and visually inspecting the MDS plot (Supplementary Fig. 1). No individuals were excluded based on this criterion. We also checked pair-wise identity by descent (IBD) and did not detect duplicate samples. Twenty-five individuals did not pass QC, thus leaving a total of 345 individuals for imputation and GWAS. After removal of individuals failing QC, we excluded all genetic variants with call rate less than 98% and all variants deviating from Hardy-Weinberg equilibrium (HWE) (P < 10^−4^).

Following QC, we performed genotype imputation on clean dataset. All SNPSs were aligned to the forward strand prior to the imputation. Genotypes were firstly pre-phased using SHAPEIT2 software^30^ and then imputed with IMPUTE2 software^31^ using 1000 Genomes (Phase3, all populations) as reference panel. We checked concordance tables to assess an overall imputation quality, mean genotype concordance in our dataset was 95,51%. We also compared the concordance rate and INFO (type 0) metric of directly typed SNPs to detect poorly genotyped SNPs or possible strand flips. Performed analysis showed that all directly genotyped SNPs used in imputation were well genotyped and correctly aligned to the forward strand. After imputation, we excluded all genetic variants with MAF less than 5%, variants with INFO metric less than 0.4 and variants with HWE P < 10^−4^. A total of 6 007 542 autosomal genetic variants passed post-imputation QC and were selected for GWAS.

### Genome-wide association analyses

We performed GWAS of thyroid volume in two groups of HT patients separately, depending on levothyroxine (LT4) therapy. There were 130 patients that were on LT4 therapy and 215 patients without LT4 therapy. Thyroid volume was firstly adjusted for covariates under standard linear regression model in R: age, gender, TSH and BSA were used as covariates in both groups of HT patients, as they are known to influence thyroid volume. We used LT4 dosage as an additional covariate in the group of HT patients that were on LT4 therapy, as LT4 therapy reduces thyroid volume^32,33^. Residuals derived from linear regression model were quantile transformed in R and then used as a new phenotype in association analyses, instead of thyroid volume. Association analyses between 6 007 542 common autosomal genetic variants and adjusted thyroid volume were performed under the linear mixed model implemented in GEMMA software which accounts for population stratification and relatedness^34^.

### GWAS meta-analysis

Individual GWAS summary results were meta-analyzed in METAL using fixed-effects inverse variance method which weights genetic variant’s effect sizes (β-coefficients) by their standard errors (SE)^35^. Manhattan plot of GWAS meta-analysis results was generated using R package qqman. Regional association plots were generated using LocusZoom software^36^. We checked cluster plots of all directly genotyped variants with P-value less than 0.1 located +/− 400 kb from top-associated variants, to eliminate possible spurious associations. Cluster genotyping plots were generated using Ilummina GenomeStudio software. For genetic variants associated with thyroid volume on the genome-wide level of significance (P < 5 × 10^−8^), we assessed the distribution of thyroid volume per genotype and generated box plots using R statistical software.

### Verification of results using immunohistochemical analysis

Thyroid tissue paraffin blocks were obtained from HT patients who are part of our CROHT biobank and control participants from “10001 Dalmatians” biobank^37^, with available genome-wide data, who underwent thyroidectomy at University Hospital of Split. Tissues were reviewed by expert pathologist for signs of HT and for confirmation of healthy (control) tissue. We analyzed expression of AATF, SMARCA2 and apoptotic marker cytochrome C (CytC) in thyroid follicles. Immunohistochemical analysis was used to test AATF/SMARCA2 and apoptotic marker expression between HT cases and controls of the same genotype i.e. if HT patients with one/two risk alleles have decreased AATF/SMARCA2 expression and increased apoptosis in comparison to controls with the same genotype. As the risk allele T of rs7212416 (AATF) has allele frequency of 85%, we were able to acquire tissues from 3 HT patients and 7 controls with TT genotypes (individuals with two risk alleles). Due to allele frequency of 50% of the risk allele T of rs10738556 (SMARCA2), there were no thyroid specimens from individuals with homozygous TT genotypes in our Biobanks, therefore, we focused on analysis of heterozygous individuals. We were able to acquire tissues from 5 HT patients and 7 controls with heterozygous CT genotypes (i.e. individuals with one risk allele).

Formalin fixed and paraffin embedded tissue sections were deparaffinized and run through process of antigen retrieval in citrate buffer. Nonspecific binding was blocked by Protein Block (Abcam, Cambridge, UK). Tissue sections were incubated with primary antibodies to AATF (Abcam, Cambridge, UK), SMARCA2 (Abcam, Cambridge, UK) and CytC (Santa Cruz Biotechnology, Santa Cruz, CA, USA) overnight at 4 °C. Staining was visualized by incubation with secondary antibodies labeled with red (donkey anti rabbit Cy3 polyclonal, Dako, Glostrup, Denmark) and green (donkey anti mouse labeled with AF488, Invitrogen, Carlsbad, CA, USA) fluorochromes. Finally, samples were counterstained with DAPI (4′,6-diamidino-2-phenylindole).

Photo-micrographs were shot by SPOT Insight digital camera (Diagnostic Instruments, USA), mounted on Olympus BX61 fluorescence microscope (Olympus, Tokyo, Japan). Camera settings were set using image acquisition software CellA® at 1360 × 1024 resolution, exposition of 1/333.3 s with noise reduction filter. Ten micro-photographs of thyroid follicles, under the magnification of 200×, were shot per slide in all of 3 fluorescent channels. Furthermore, florescence intensity histograms were acquired for red and green fluorescence channels in ImageJ software (NIH, Bethesda, MD, USA). Region of positive signal was determined by using the slides stained with secondary antibodies only, thus quantifying the autofluorescence and fluorescence due to unspecific binding of secondary antibodies. Region of positive signal was defined as the one that excluded 99.99% of the signal obtained from florescence intensity histograms of slides stained with secondary antibodies only. Expression of AATF and SMARCA2 was quantified as the area under the curve (AUC) of florescence intensity histograms. On the other hand, since CytC release from mitochondria into cytoplasm is a marker of apoptosis, unlike its expression, we quantified it as a percentage of micro-photograph area covered by positive signal. AUCs and their interval estimates were calculated by using AUC analysis routine in GraphPad Prism 8.0 software (Graph Pad, La Jolla, CA, USA). Statistical significance and effect sizes as well as respective 95%CI were calculated by t-test with Welch correction (Graph Pad, La Jolla, CA, USA). For comparisons of percentage of area covered by positive signal t-test was used.

## Results

### Clinical characteristics of HT patients

Clinical characteristics of HT patients divided in two sets, depending on LT4 therapy, are summarized in Table 1. HT patients without LT4 therapy have mean TSH levels just slightly above the upper reference values for our population, while their median T3 and T4 levels are in normal reference ranges. Expectedly, hormone values of patients without therapy reflect the fact that these patients are newly diagnosed or in follow-up with preserved thyroid function. HT patients that are on LT4 therapy have significantly lower TSH levels (P = 0.0001), and significantly higher T4 (P < 0.0001) and fT4 (P < 0.0001) levels, however their T3 and T4 levels are in reference ranges for our population, suggesting that their LT4 supplementation is appropriate. Male to female ratio is in accordance to expected distribution of HT according to the gender, with higher incidence in females^38^. Additionally, HT patients that are on LT4 therapy have significantly lower thyroid volume (P = 0.0024). There are no significant differences between two groups of HT patients in age, gender, BSA, and T3 levels. We also do not observe differences in median TgAb/TPOAb levels between two groups of patients.

**Table 1:** Clinical characteristics of HT patients with and without levothyroxine (LT4) therapy.

Variable	NT (N = 215)	OT (N = 130)	P
	Median (Q1–Q3)	Median (Q1–Q3)	
Age, years	37.5 (27.8–45.6)	41.3 (30.2–49.5)	0.0623^a^
Thyroid volume, cm^3^	10.6 (7.8–14.5)	8.7 (6.6–12.2)	0.0024^a^
BSA, m^2^	1.8 (1.69–1.92)	1.82 (1.69–1.94)	0.6892^a^
TSH, mIU/L	3.77 (1.97–6.03)	2.46 (1.34–4.14)	0.0001^a^
T3, nmol/L	1.6 (1.4–1.8)	1.7 (1.4–1.8)	0.5275^a^
T4, nmol/L	99.1 (85.8–115)	113 (95.8–125.7)	<0.0001^a^
fT4, pmol/L	11.9 (10.1–12.8)	12.7 (11.75–14.1)	<0.0001^a^
TgAb, IU/ml	124 (31.7–441)	158 (50.5–419.8)	0.6158^a^
TPOAb, IU/ml	219 (23.4–670.5)	174.5 (47.9–628.2)	0.9387^a^
LT4 dosage, μg	/	75 (50–100)	/
	**N (%)**	**N (%)**	
Gender			0.1514^b^
Males	10 (5%)	11 (8%)	
Females	205 (95%)	119 (92%)	

NT-HT patients that are not taking LT4 therapy, OT-HT patients that are on LT4 therapy.

Q1-first quartille, Q3-third quartile, P-p-value.

^a^Mann-Whitney-U test, ^b^ χ^2^-test.

### GWAS meta-analysis

**Figure 1:**
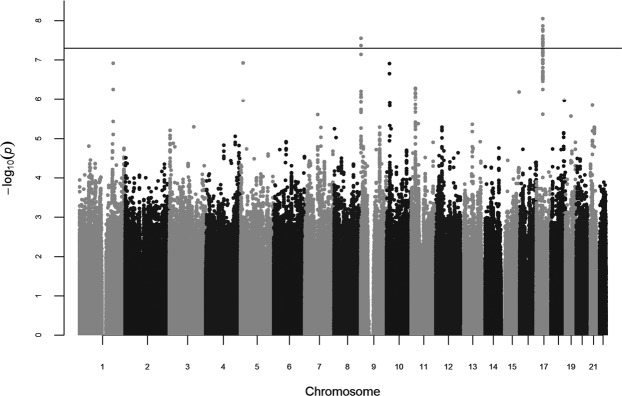
Manhattan plot of GWAS meta-analysis results. For each analyzed genetic variant, the x-axis shows chromosomal position, while y-axis shows the −log_10_(P) value. The horizontal line indicates the genome-wide significance threshold of P = 5 × 10^−8^.

**Figure 2:**
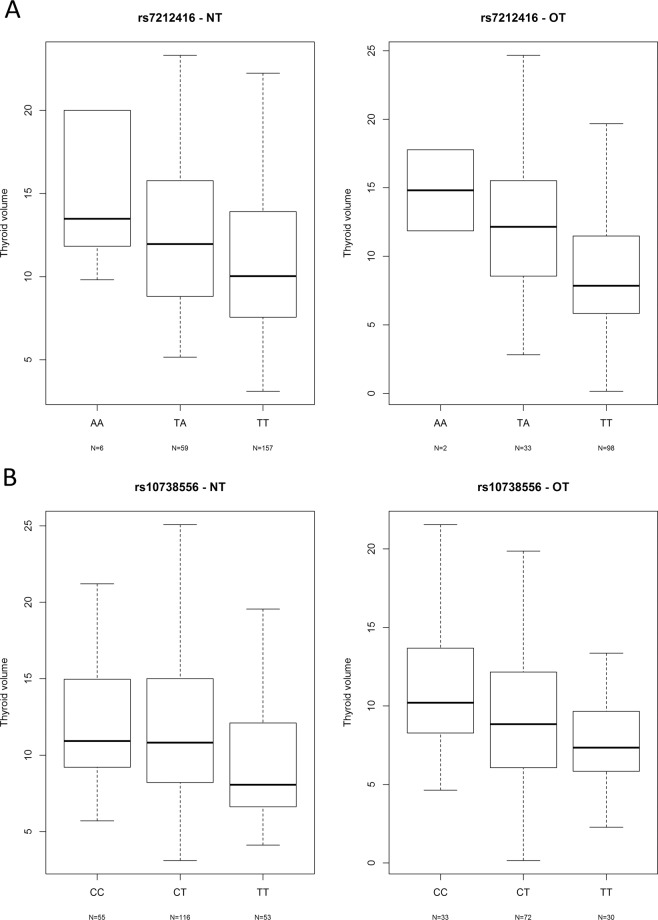
Box plots of distribution of thyroid volume per genotype in both groups of HT patients, depending on levothyroxine (LT4) therapy, for two genome-wide significant hits: rs7212416 (**A**) and rs10738556 (**B**). NT- HT patients that are not taking LT4 therapy, OT- HT patients that are on LT4 therapy.

We identified two genome-wide significant hits in this first GWAS meta-analysis of thyroid volume in HT patients (Fig. 1). The most associated SNPs representing each signal are rs7212416 inside apoptosis-antagonizing transcription factor *AATF* (P = 8.95 × 10^−9^, β = −0.589, SE = 0.102 for allele T) and rs10738556 located 7 kb from chromatin-remodeling *SMARCA2* (P = 2.83 × 10^−8^, β = −0.44, SE = 0.079 for allele T) (Table 2). Regional association plots of these genetic variants are shown in Supplementary Fig. 2. Box plots of distribution of thyroid volume per genotype for both genetic variants are shown in Fig. 2.

**Table 2:** The most associated genetic variants from GWAS meta-analysis of thyroid volume in HT patients.

Chr	Position	SNP	EA	OA	INFO score	Gene/nearest gene	NT (N = 215*)				OT (N = 130*)				Meta-analysis (N = 345*)		
							EAF	β	SE	P	EAF	β	SE	P	β	SE	P
17	35332740	rs7212416	T	A	0,956	*AATF*	0,84	−0,581	0,126	6,45E-06	0,86	−0,605	0,177	8,54E-04	−0,589	0,1024	8,95E-09
9	2007878	rs10738556	T	C	0,895	7 kb from *SMARCA2*	0,50	−0,430	0,101	3,24E-05	0,48	−0,458	0,128	4,87E-04	−0,440	0,079	2,83E-08
5	13830037	rs201666766	A	T	0,533	*DNAH5*	0,93	−1,087	0,251	2,23E-05	0,91	−0,990	0,325	2,87E-03	−1,051	0,199	1,21E-07
1	184912127	rs170884	C	A	0,867	*FAM129A*	0,67	0,249	0,115	3,17E-02	0,64	0,700	0,127	2,02E-07	0,452	0,085	1,22E-07
10	16311536	rs4747268	A	C	0,98	167 kb from *PTER*	0,17	−0,517	0,130	9,25E-05	0,14	−0,575	0,165	6,81E-04	−0,539	0,102	1,26E-07
11	23347265	rs193145729	G	C	0,99	93 kb from *MIR8054*	0,89	0,559	0,140	8,60E-05	0,86	0,509	0,168	2,95E-03	0,538	0,107	5,25E-07
15	99236276	rs11247367	G	A	0,643	*IGF1R*	0,90	0,775	0,193	8,06E-05	0,92	0,809	0,276	4,03E-03	0,786	0,158	6,59E-07
11	23171685	rs10767013	G	T	0,997	269 kb from *MIR8054*, 290 kb from *CCDC179*	0,49	−0,275	0,092	3,24E-03	0,48	−0,510	0,120	4,14E-05	0,362	0,073	7,44E-07

NT-HT patients that are not taking levothyroxine (LT4) therapy, OT-HT patients that are on LT4 therapy, Chr-chromosome, EA-effect allele, OA-other allele.

EAF-effect allele frequency, β-SNP effect size, SE-standard error, P-p-value. Positions are based on the GRCh 37 build. All β (SE) values are calculated for effect allele.

*Number of HT patients that passed quality control and were included in GWAS analyses.

We identified additional six suggestively associated loci with thyroid volume (P < 10^−6^) (Fig. 1): rs201666766 inside *DNAH5* (P = 1.21 × 10^−7^, β = −1.051, SE = 0.199 for allele A), rs170884 inside *FAM129A* (P = 1.22 × 10^−7^, β = 0.452, SE = 0.085 for allele C), rs4747268 close to *PTER* (P = 1.26 × 10^−7^, β = −0.539, SE = 0.102 for allele A), rs193145729 close to *MIR8054* (P = 5.25 × 10^−7^, β = 0.538, SE = 0.107 for allele G), rs11247367 inside *IGF1R* (P = 6.59 × 10^−7^, β = 0.786, SE = 0.158 for allele G) and rs10767013 between *MIR8054* and *CCDC179* (P = 7.44 × 10^−7^, β = 0.362, SE = 0.073 for allele G) (Table 2). Regional association plots of these genetic variants are shown in Supplementary Fig. 3.

### Immunohistochemical analysis of AATF and SMARCA2

**Figure 3:**
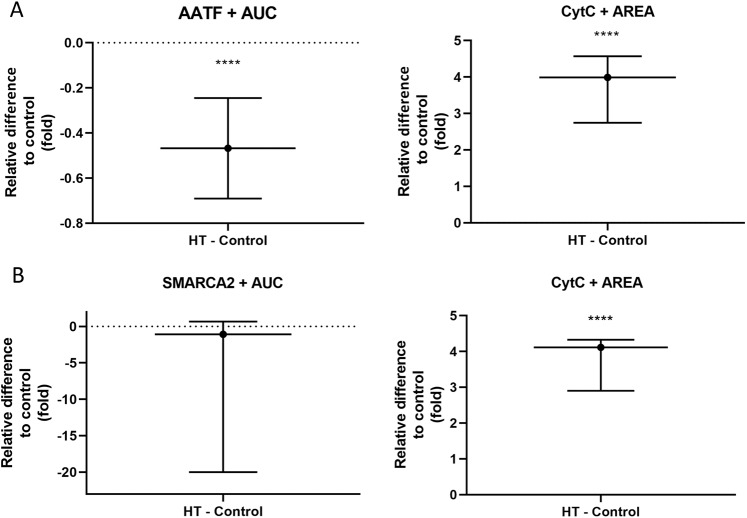
Differences in expression of (**A**) AATF and CytC between HT patients (n = 3) and controls (n = 5) with rs7212416 TT genotypes (**B**) SMARCA2 and CytC in HT patients (n = 5) and controls (n = 7) with rs10738556 CT genotypes. Data is represented as mean and 95% CI of fold change in relative difference between groups, normalized to control group. ****p < *0.0001.*

In order to examine expression patterns on protein level, we measured expression of AATF along with apoptosis in thyroid tissues of samples with rs7212416 TT genotypes. Patients with HT had significant 0.46-fold decrease in AATF levels when compared to control patients (95% CI: −0.69 to −0.24, p < 0.0001). This was accompanied by significant increase in apoptosis by 3.99-fold in comparison to control samples (95% CI: 2.74 to 4.57, p = 0.0001), as shown in Fig. 3A and Supplementary Fig. 4.

We also analyzed expression of SMARCA2 and apoptosis in thyroid tissue of samples with rs10738556 CT genotypes. SMARCA2 levels were decreased by 1.07-fold in HT group in comparison to control group, however without reaching statistical significance (95% CI: −19 to 0.67, p = 0.5876), whereas we observe significant increase in apoptosis by 4.11-fold in HT group (95% CI: 2.9 to 4.32, p < 0.0001) (Fig. 3B, Supplementary Fig. 4).

## Discussion

We have identified two genome-wide significant loci with thyroid volume of HT patients, one in apoptosis-antagonizing transcription factor *AATF* (also known as *Che-1*) and other near chromatin remodeling *SMARCA2*, associated with transcriptional regulation. Additionally, we have identified six loci suggestively associated with thyroid volume, of which two, *IGF1R* and *FAM129A*, play a role in apoptosis, thyroid morphogenesis and thyroid carcinomas, respectively. Identification of mentioned genes with clear connection to apoptosis is particularly interesting as apoptosis of thyrocytes affects the mass of thyroid gland and is considered to be the landmark of HT^8–12^. We additionally performed immunohistochemical analysis of expression levels of AATF, SMARCA2 and apoptotic marker in thyroid tissues of HT patients and controls to further explore involvement of identified proteins in pathophysiological processes of thyroid gland of HT patients.

Here, we observe a negative effect of the major allele T (allele frequency 85%) of the most associated *AATF* genetic variant, rs7212416, on thyroid volume. Patients with major allele on this SNP have decreased thyroid volume (Table 2, Fig. 2A). Our hypothesis is that in the microenvironment of thyroid gland of HT patients, underlying genetic variants may affect AATF activity and possibly regulate the magnitude of apoptosis. As AATF has anti-apoptotic role, increased apoptotic activity in thyroid gland of HT patients might be modulated by decreased activity of AATF, due to its lower expression or ubiquitin-dependent degradation^39^. We additionally performed immunohistochemical analysis in which we were able to demonstrate that HT patients (homozygous for AATF rs7212416 risk allele T), have significantly decreased AATF expression and significantly increased apoptosis in comparison to controls with the same genotype (Fig. 3A, Supplementary Fig. 4). These results provide further support of involvement of AATF in HT pathophysiology. However, additional experiments need to be performed to drive conclusion about association of identified SNP with AATF expression levels and apoptosis.

We also observe in GWAS analysis that the effect is stronger (absolute β value) in the group of HT patients that are in progressed stage of disease (overt hypothyroidism and on LT4 therapy) in comparison to newly diagnosed HT patients without LT4 therapy (Table 2), which is in line with the hypothesis that thyroid atrophy is a proxy for underlying apoptosis and is more pronounced in later stages of HT.

Beside an anti-apoptotic role, AATF has several other versatile, but correlative roles in transcriptional regulation, induction of cell proliferation, cell cycle checkpoint control and response to DNA damage through activation of p53^40–44^. Bruno *et al*. suggested interesting role of AATF as a novel effector in the regulation of p53 pathway^41^. In this model, DNA damage-induced phosphorylation and stabilization of AATF, set off cellular arrest by increasing transcription of p53 and p21^41^. An anti-apoptotic role of AATF is further suggested because activation of DNA damage checkpoints by AATF promotes DNA repair and cell survival^41^, whereas in the case of extensive DNA damage, a cell is destined for apoptosis and AATF is targeted for degradation^39^. Similarly, experimental silencing of AATF resulted with decreased cell proliferation and promotion of apoptosis^42,45^. Additionally, upon AATF silencing, pro-apoptotic genes, including Fas and FasL, were up regulated, while multiple anti-apoptotic genes were down-regulated^45^. Since the apoptosis via Fas death receptor is the hallmark of HT^10^, involvement of AATF in Fas/FasL signaling is compelling.

As we have previously performed GWAS study of 405 HT cases and 430 controls^26^, we were able to check allele frequencies of the most associated AATF genetic variant (rs7212416) in both groups. We do not observe difference in allele frequency distribution of this SNP between HT patients and controls implying that an effect of this SNP on thyroid volume may be triggered by pathologic conditions in thyroid of HT patients. A further indication of suggested mechanism is demonstrated by our immunohistochemical analysis where we found significantly decreased AATF levels and increased apoptosis in thyroid follicles of HT patients in comparison to controls with the same rs7212416 TT genotype. We therefore propose that SNP effect may be secondary to inflammatory events in the thyroid of HT patients where inflammatory cytokines initiate autoimmunity and play regulative roles in expression of apoptotic signaling^46,47^. Studies have shown that thyrocytes, alongside immune cells, contribute to secretion of proinflammatory cytokines of Th1 type that stimulate autocrine/paracrine apoptosis induction of thyrocytes^48^. However, as already marked, true association between identified genetic variant and apoptosis needs to be analyzed in additional tests.

Interestingly, a genomic region encompassing AATF has already been strongly associated with chemokine macrophage inflammatory protein 1b levels (MIP-1-beta)^49^, which is involved in cytokine signaling in immune system^50^. This chemokine has already been associated with autoimmune pathology of adrenal gland^51^. Furthermore, MIP-1-beta has also been linked to Fas-FasL mediated apoptosis^52^. As chemokines, together with Th1 cytokines, are considered the main regulators of autoimmune process in HT, a finding of association of the same genomic region with MIP-1-beta and regulation of thyroid volume is intriguing and provides multiple lines of evidence of involvement of this genomic region with apoptosis.

This study presents yet another important link between our two major hits, *AATF* and *SMARCA2*. A transcriptional regulator SMARCA2, also known as hBRM, is a component of SWI/SNF family of proteins that changes chromatin structure through altering DNA-histone contacts (www.genecards.org). hSWI/SNF chromatin remodelers have already been linked to apoptosis^53,54^. Overexpression of SMARCA2 has been associated with decrease in apoptosis of ovarian cancer cells^55^ and pancreatic cancer cells^56^. On the other hand, SMARCA2 is found to be cleaved during apoptosis^57^ and its truncated levels correlate with cell damage and apoptosis after virus infection^58^. It has been shown that SMARCA2 also interacts with HDAC1^59^, the deacetylase known for its role in regulation of the cell cycle progression through Rb binding that is inhibited by AATF^60^. In brief, Bruno *et al*. have proven that AATF directly inactivates HDAC1/Rb/E2F1 transcriptional inhibition by competition with HDAC1 for the Rb binding site, thus inducing cellular growth and proliferation. On the other hand, a glucocorticoid receptor-induced apoptosis is found to be dependent on Rb and SMARCA2^61^. From our results, we can speculate that both AATF and SMARCA2, through common interaction with HDAC1 and Rb, could be involved in the regulation of transcription that blocks cell proliferation and subsequently drives thyrocytes to apoptosis in thyroid gland of HT patients.

We also demonstrate that HT patients (heterozygous for SMARCA2 rs10738556) have decreased SMARCA2 expression, albeit without reaching statistical significance, and significantly increased apoptosis in comparison to controls (Fig. 3B, Supplementary Fig. 4). We assume that due to analysis of heterozygous individuals i.e. individuals with only one risk allele, we observed a weaker decrease in SMARCA2 expression. Although these results suggest involvement of SMARCA2 in molecular processes specific to HT, more tests need to be performed to give definite conclusion on association of identified genetic variant with differential SMARCA2 expression and induction of apoptosis.

Furthermore, *SMARCA2* is involved in the same pathway as our next suggestively associated locus, *IGF1R*. They both belong to hepatocellular carcinoma (HCC) pathway that has in turn been associated with abnormal thyroid hormone signaling^62^ further linking our genetic variants with thyroid dysfunction. IGF1R has pleiotropic roles in humans, among which two roles are relevant for our study. Firstly, it is an anti-apoptotic marker and, secondly, it has a direct role in functionality of thyroid gland. In *in vitro* conditions, IGF1R works together with TSH in stimulating thyrocyte growth and is actually found to be essential for regulation of thyroid function and TSH-stimulated goitrogenesis^63^. Important role in maintaining homeostasis and normal thyroid morphogenesis was further demonstrated in animal model^64^. These data suggest an important role of IGF1R with thyroid growth morphogenesis thus providing a concrete support for our finding of association of this gene with thyroid volume. We believe that this is a true candidate gene for maintaining thyroid volume albeit our study was not powerful enough to detect its association on a genome-wide level.

Finally, we bold out our second suggestively associated locus, *FAM129A*, also known as *C1orf24* and *NIBAN*, as it was also found to act in anti-apoptotic manner, in regulation of p53-mediated apoptosis^65^. FAM129A is highly expressed in carcinomas including thyroid carcinomas^66,67^ and is used as one of several markers that may differentiate benign from malignant thyroid lesions, therefore, it is considered to be the best predictor for thyroid cancer^68–71^. FAM129A silencing in thyroid carcinoma cell lines directly leads to apoptosis, cell migration inhibition and cell cycle progression^70^. Finally, immunohistochemical staining showed that FAM129A, beside in thyroid tumors, is weekly expressed in subsets of scattered cells with oxyphilic cell metaplasia in HT and not in normal thyroid tissue^71^. Taken together, the current knowledge on this gene and our results, imply the role of FAM129A in the apoptosis regulation in thyroid tissue of HT patients.

Beside the role of FAM129A in thyroid cancer, the loss of SMARCA2 was also found to be associated with thyroid cancer, particularly with anaplastic thyroid cancer^72^. Given the fact that some studies report an increased incidence of well-differentiated thyroid cancer in chronic autoimmune thyroiditis^73,74^, it is possible that there is a genetic link between HT and thyroid cancer, i.e. that changes in expression of SMARCA2 and FAM129A in HT can predispose to development of some forms of thyroid cancer. If this holds true, then SMARCA2/FAM129A could be potential biomarkers for stratifying HT patients who maybe of higher risk of developing thyroid cancer. However, more research using prospective studies with full genetic profiling is needed to test this hypothesis, as our study was not designed to give answer on genetic correlation between HT and thyroid carcinomas.

Further, we have identified another four suggestively associated variants located inside or near *DNAH5*, *PTER* and *MIR8054*. Genetic variants inside these genomic regions were previously associated with number of traits, such as bacterial meningitis (DNAH5 region)^75^, hepatitis B (*PTER* region)^75^, cancer (*MIR8054* region)^76^, allergic rhinitis (*MIR8054* region)^77^.

Finally, we reflect on limits of our study, the main one being the sample size. Although the sample size of 345 individuals, as used in our study, is considered to be small dataset for GWAS studies, we have carefully selected our phenotype and study population to increase genetic effect sizes and thus the power of our study. It is known that a thoughtful selection of investigated phenotypes, for example for the disease severity, may enrich for genetic causes^78^. Thyroid volume in HT patients is considered to change over the course of disease and it is smaller in patients with prolonged disease duration, representing tissue degradation^5^. This is exactly what we observe in our cohort, individuals with prolonged disease and in overt hypothyreosis (those that are taking LT4 therapy), have smaller thyroid volumes than newly diagnosed individuals (Table 1). We have used this information to increase the power of our study and analyzed thyroid volume separately in two subsets of HT patients, depending on LT4 therapy, as these two subsets reflect the severity of disease, and then meta-analyzed across. Finally, our initial hypothesis was that a change in size of thyroid tissue may be seen as a proxy for change in biological activity of thyroid but also as manifestation of underlying apoptotic processes. The results of our study validate the design of our GWAS analysis. However, as we do not have replication cohort, and to our knowledge, there is currently no other cohort of HT patients with available GWAS data and thyroid dimensions, it is very important to replicate and confirm our findings in other genetic and functional studies. Furthermore, we found significantly decreased AATF and indication of decreasing SMARCA2 expression levels accompanied by significantly increased apoptosis in HT patients with two or one risk allele, respectively. Our study is not designed to provide direct evidence that identified genetic variants are associated with decrease in AATF/SMARCA2 expression and increase in apoptosis, therefore, for confirmation of our hypothesis, a further study on causality between association of genetic variants with expression of AATF/SMARCA2 and apoptosis in thyroid cell lines is needed. Additionally, due to lack of available thyroid tissue, we could not evaluate expression levels of AATF/SMARCA2 and apoptosis in individuals among three different genotypes.

In summary: (a) our GWAS study of thyroid volume in HT patients identified association of two genetic variants in AATF/SMARCA2, thus implying that regulation of thyroid volume of patients with HT has a genetic component; (b) further analysis of expression patterns showed differential expression levels of AATF and SMARCA2 between HT cases and controls of the same genotype accompanied by significantly increased apoptosis in HT patients, further suggesting their role in pathophysiological processes of thyroid gland of HT patients. Additional clarification of mechanisms through which identified genetic variants exhibit their effect is needed. Based on results of our study and function of associated proteins, we speculate that these genetic variants may display their effect under specific pathophysiologic conditions of thyroid gland of HT patients, possibly through modulation of AATF/SMARCA2 expression levels and induction of apoptosis. Our study provides suggestive evidence of involvement of six more loci in regulation of thyroid volume, of which *IGF1R* and *FAM129A* have also been involved in apoptosis and thyroid gland functionality. If manipulation of apoptosis would be possible in HT patients, as suggested to be one of therapeutic strategies for autoimmune diseases^79^, then these proteins, especially AATF, could be good therapeutic target candidates for testing the prevention of further thyroid damage in HT patients. In conclusion, we identified two highly plausible genetic loci, *AATF* and *SMARCA2*, which seem to be involved in determining the thyroid volume in HT. The results of our study significantly add to the current knowledge of disturbed biological mechanisms in thyroid gland of HT patients and pathogenesis of HT on molecular level.

## Supplementary information

Supplementary Material.

## Data Availability

Genotype data are deposited in Croatian Digital Academic Archives and Repositories (DABAR) and are available following this link: https://urn.nsk.hr/urn:nbn:hr:171:066445.
