# Supplementary material for: AATF and SMARCA2 are associated with thyroid volume in Hashimoto’s thyroiditis patients
Source: Sci Rep. 2020 Feb 4;10:1754. doi: 10.1038/s41598-020-58457-x (PMC7000742; doi:10.1038/s41598-020-58457-x)
Supplement: Supplementary file 1 — Supplementary Material. [file 41598_2020_58457_MOESM1_ESM.pdf]

Supplementary Material

Supplement to: "AATF and SMARCA2 are associated with thyroid volume in Hashimoto's thyroiditis patients".

Authors: Luka Brčić[a], Ana Barić[b], Benjamin Benzon[c], Marko Brekalo[b], Sandra Gračan[b], Dean Kaličanin[a], Veselin Škrabić[d], Tatijana Zemunik[a], Maja Barbalić[a], Ivana Novak[e], Valdi Pešutić Pisac[f], Ante Punda[b], Vesna Boraska Perica[a]

[a]Department of Medical Biology, University of Split, School of Medicine, Split, Croatia

[b]Department of Nuclear Medicine, University Hospital of Split, Split, Croatia

[c]Department of Neurosciences, University Hospital of Split, Split, Croatia

[d]Department of Pediatrics, University Hospital of Split, Split, Croatia

[e]Department of Immunology and Medical Genetics, University of Split, School of Medicine, Split, Croatia

[f]Clinical Department of Pathology, Forensic Medicine and Cytology, University Hospital of Split, Split, Croatia

Contents

Supplementary Figure 1 2
Supplementary Figure 2 4
Supplementary Figure 3 5
Supplementary Figure 4 8

A

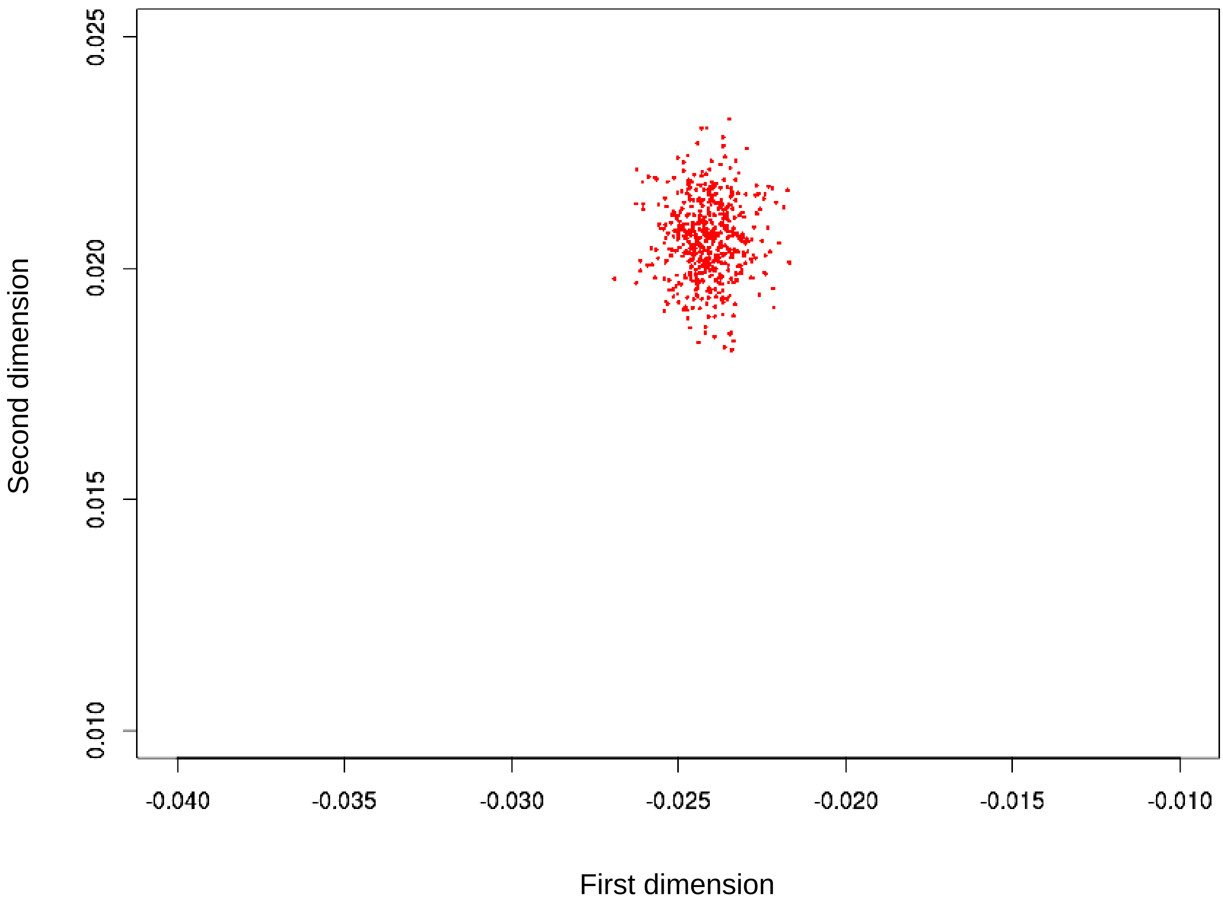
0.025
0.020
0.015
0.010
Second dimension
-0.040
-0.035
-0.030
-0.025
-0.020
-0.015
-0.010
First dimension

B

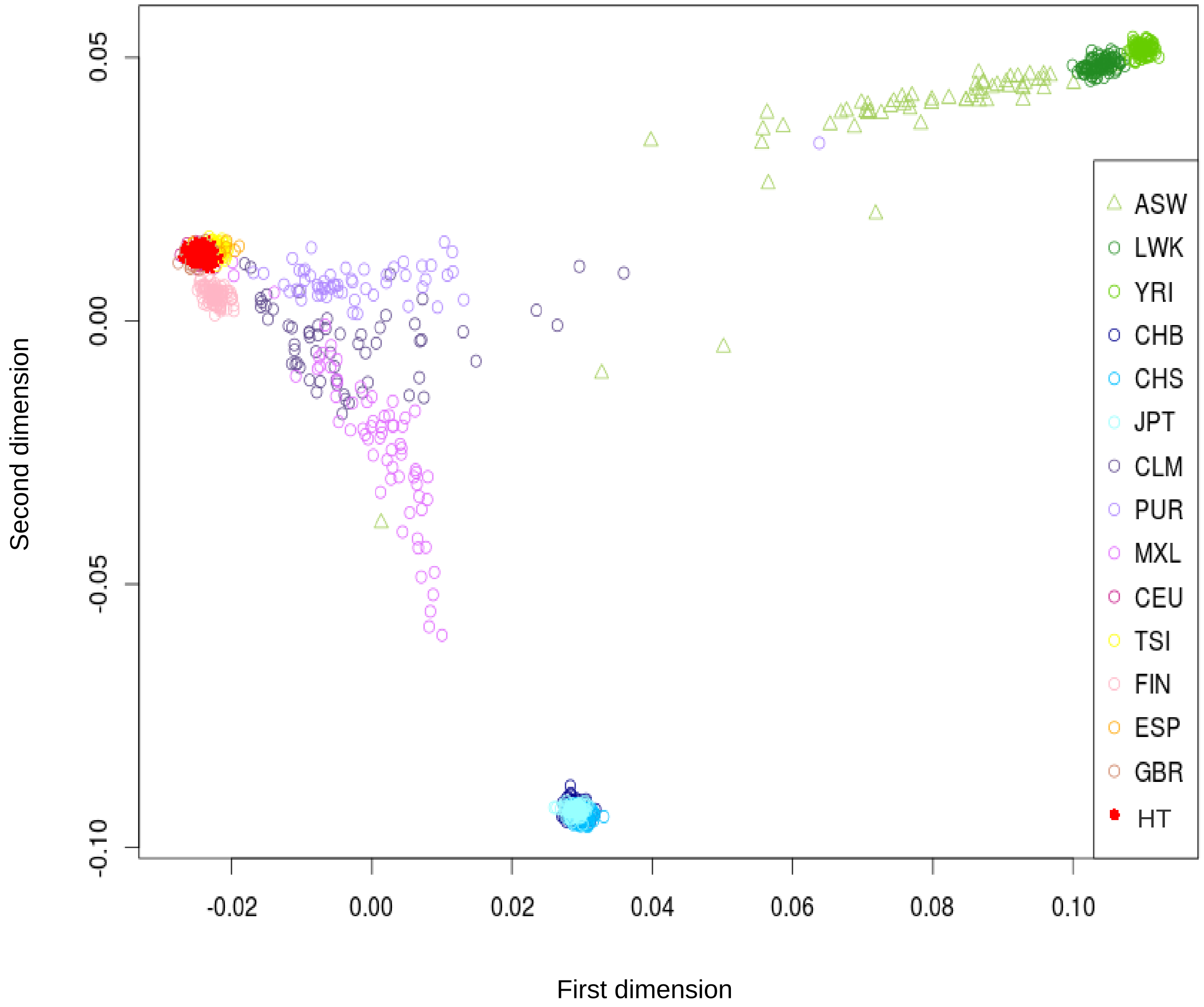

Supplementary Figure 1. (A) Multidimensional scaling (MDS) plot for all HT patients included in study. **(B)** MDS plot for all HT patients included in study along with MDS plots for other world populations from 1000 Genomes project.

Legend: **ASW** - Americans of African Ancestry in SW USA, **LWK** - Luhya in Webuye, Kenya, **YRI** - Yoruba in Ibadan, Nigeria, **CHB** - Han Chinese in Beijing, China, **CHS** - Southern Han Chinese, **JPT** - Japanese in Tokyo, Japan, **CLM** - Colombians from Medellin, Colombia, **PUR** - Puerto Ricans from Puerto Rico, **MXL** - Mexican Ancestry from Los Angeles USA, **CEU** - Utah Residents (CEPH) with Northern and Western European Ancestry, **TSI** - Toscani in Italia, **FIN** - Finnish in Finland, **ESP** - Iberian Population in Spain, **GBR** - British in England and Scotland, **HT** – HT patients from Split (Croatia) included in study.

A

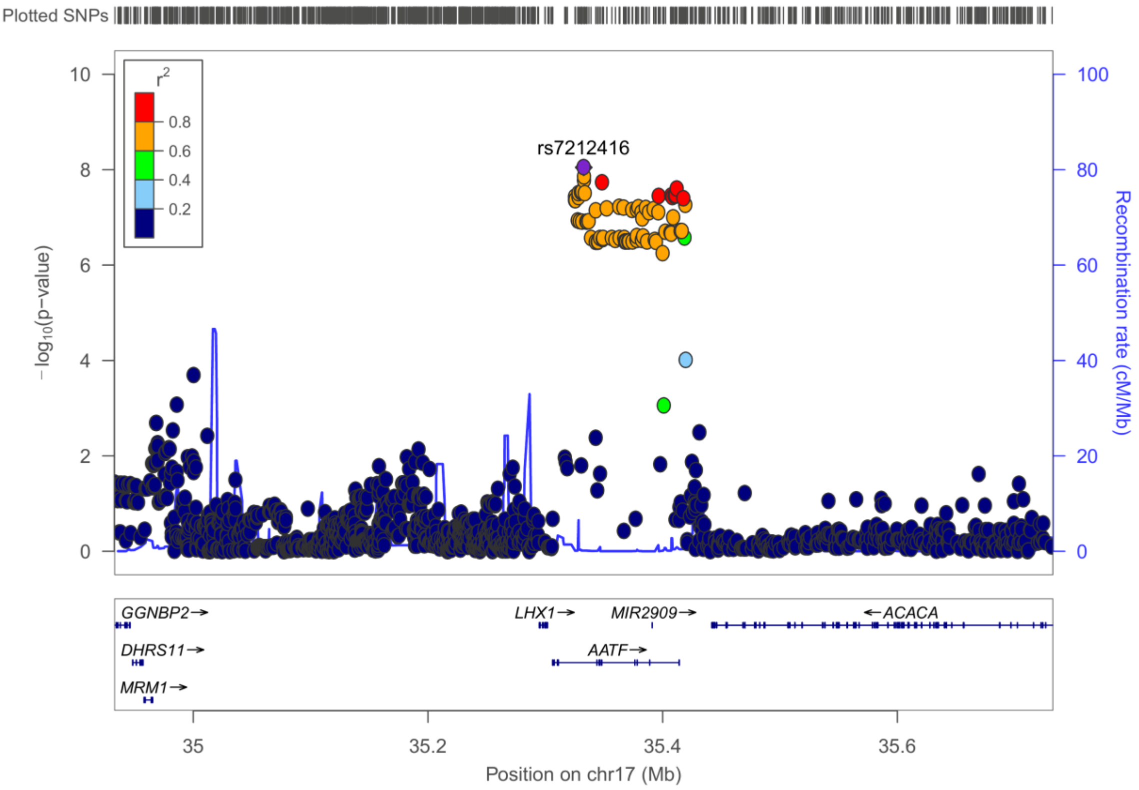

B

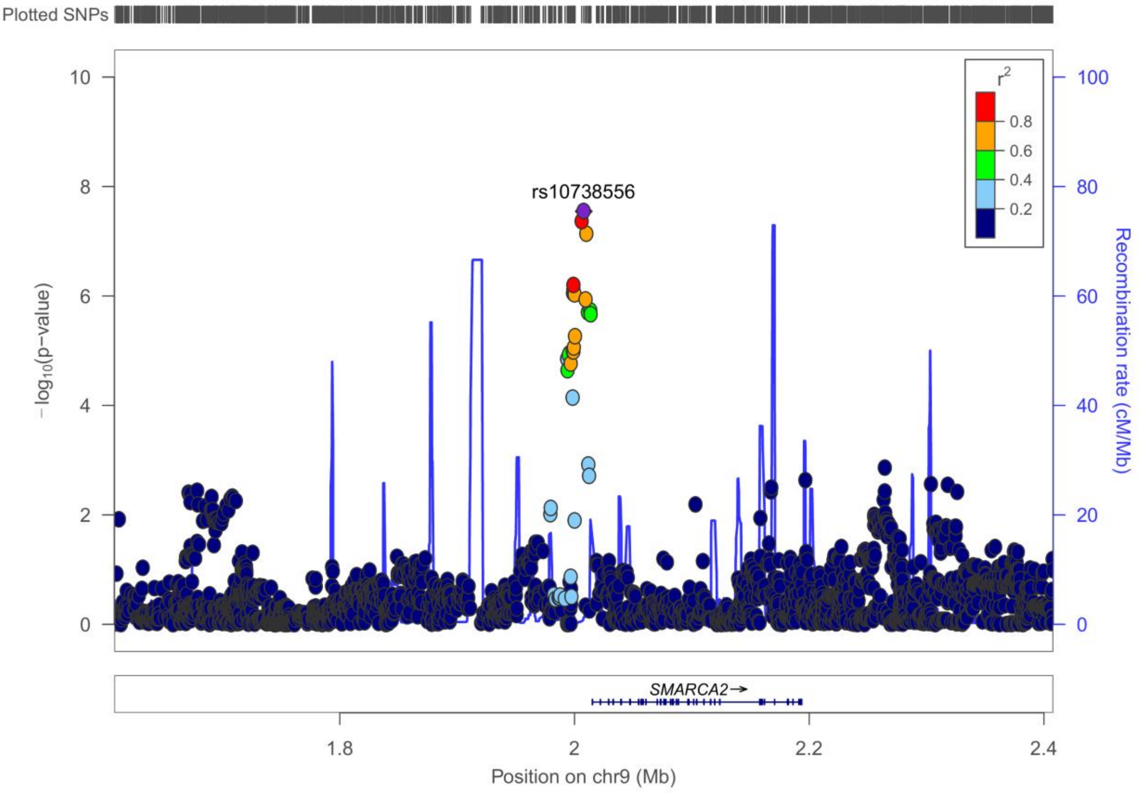

Supplementary Figure 2. Regional association plots for two genome-wide significant hits: rs7212416 (A) and rs10738556 (B).

A

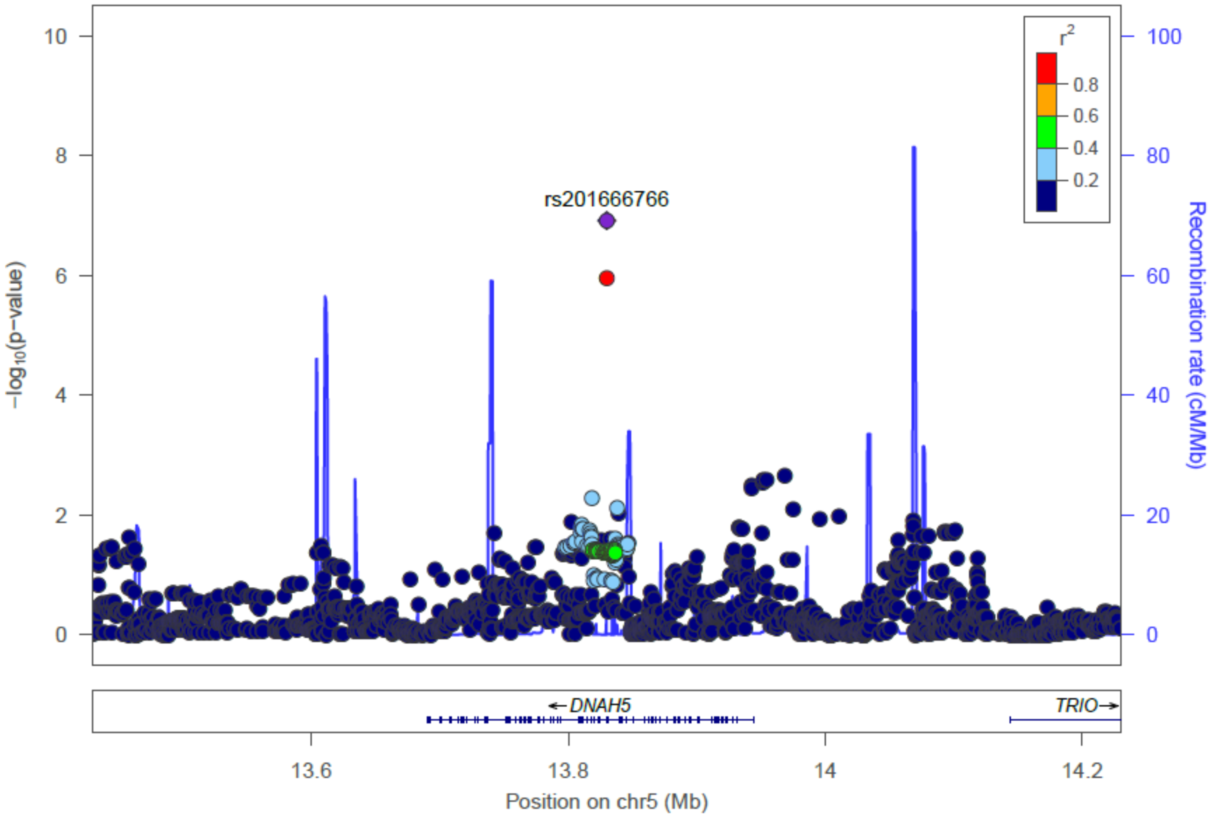

r2
0.8
0.6
0.4
0.2
rs201666766
−log10(p-value)
Recombination rate (cM/Mb)
DNAH5
TRIO
13.6
13.8
14
14.2
Position on chr5 (Mb)

B

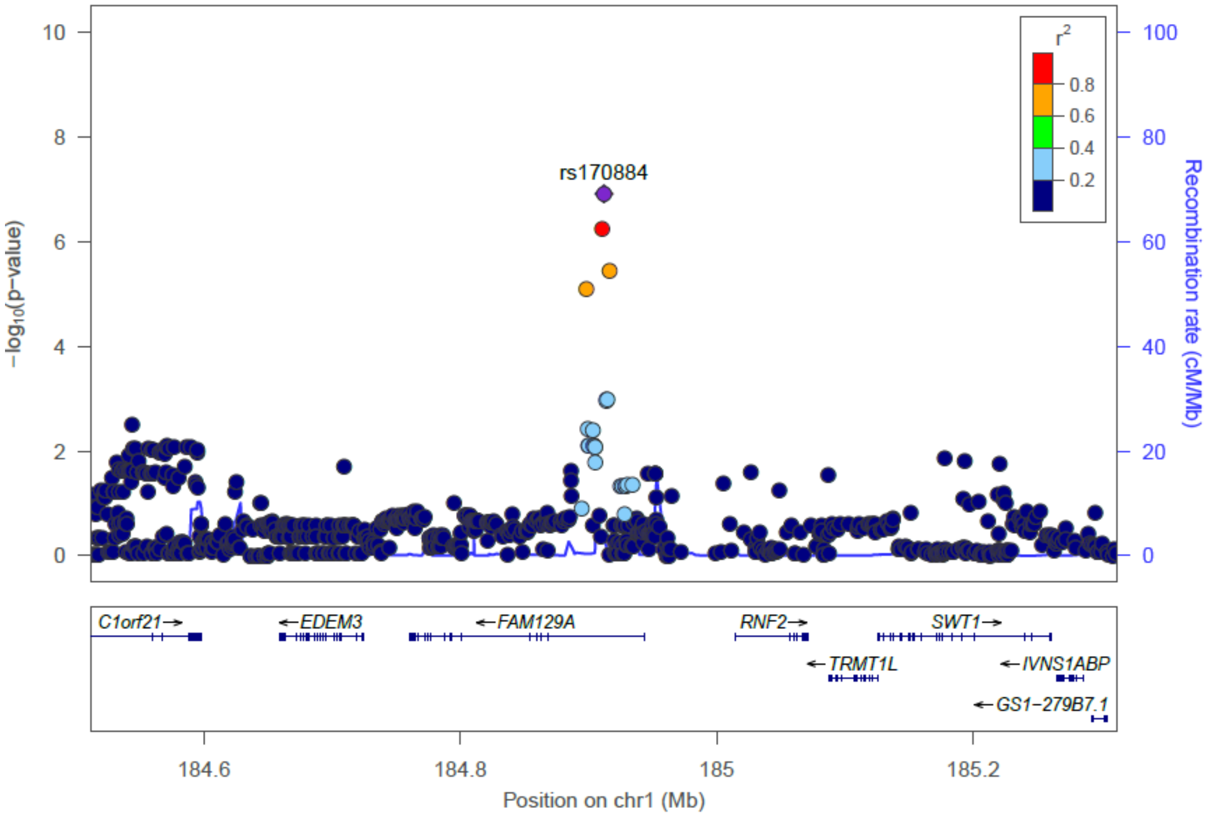

r2
0.8
0.6
0.4
0.2
rs170884
−log10(p-value)
Recombination rate (cM/Mb)
C1orf21
EDEM3
FAM129A
RNF2
SWT1
TRMT1L
IVNS1ABP
GS1−279B7.1
184.6
184.8
185
185.2
Position on chr1 (Mb)

C

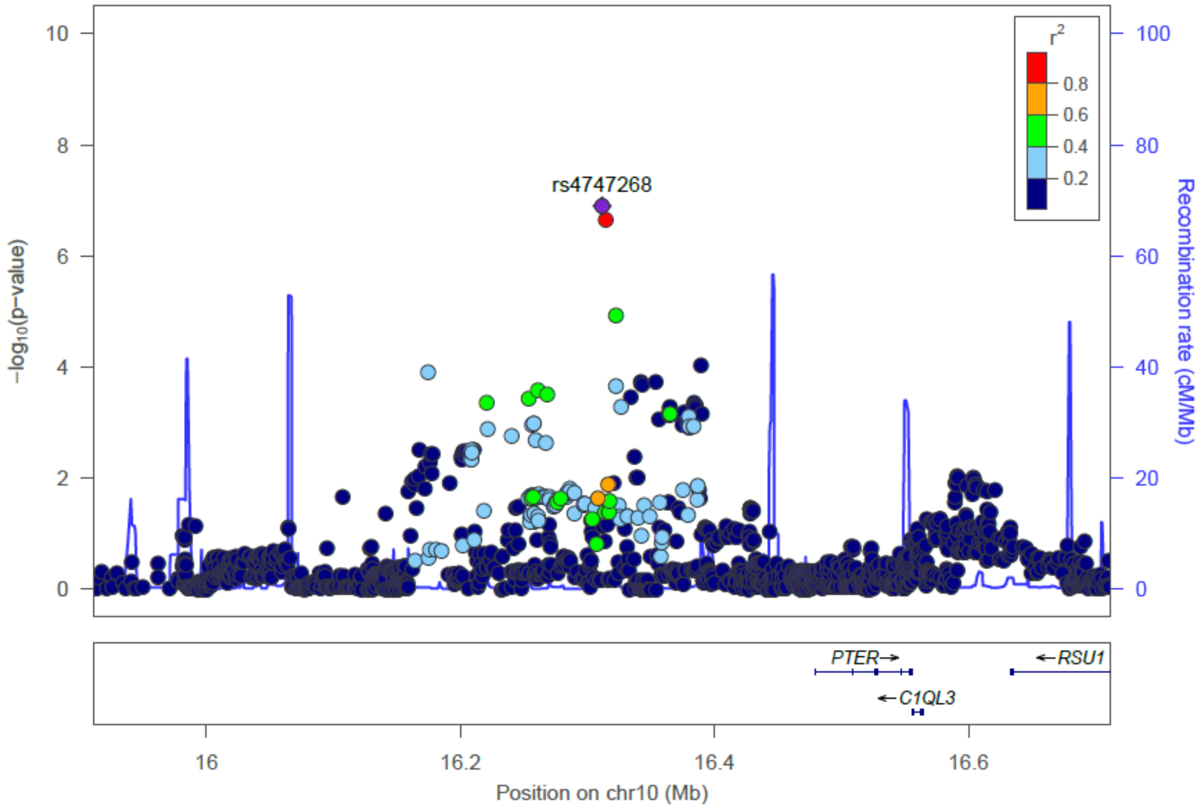

rs4747268
r2
0.8
0.6
0.4
0.2
−log10(p−value)
Recombination rate (cM/Mb)
PTER→
←RSU1
←C1QL3
Position on chr10 (Mb)

D

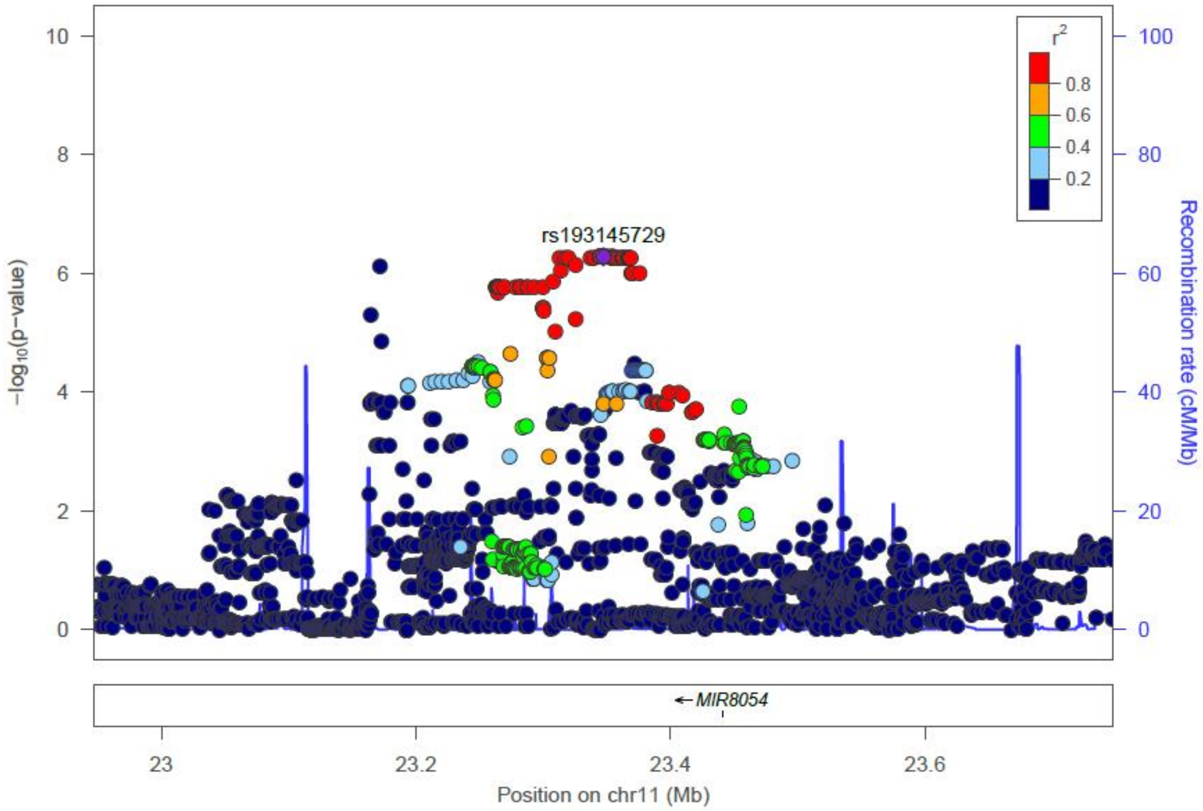

rs193145729
r2
0.8
0.6
0.4
0.2
−log10(p−value)
Recombination rate (cM/Mb)
←MIR8054
Position on chr11 (Mb)

E

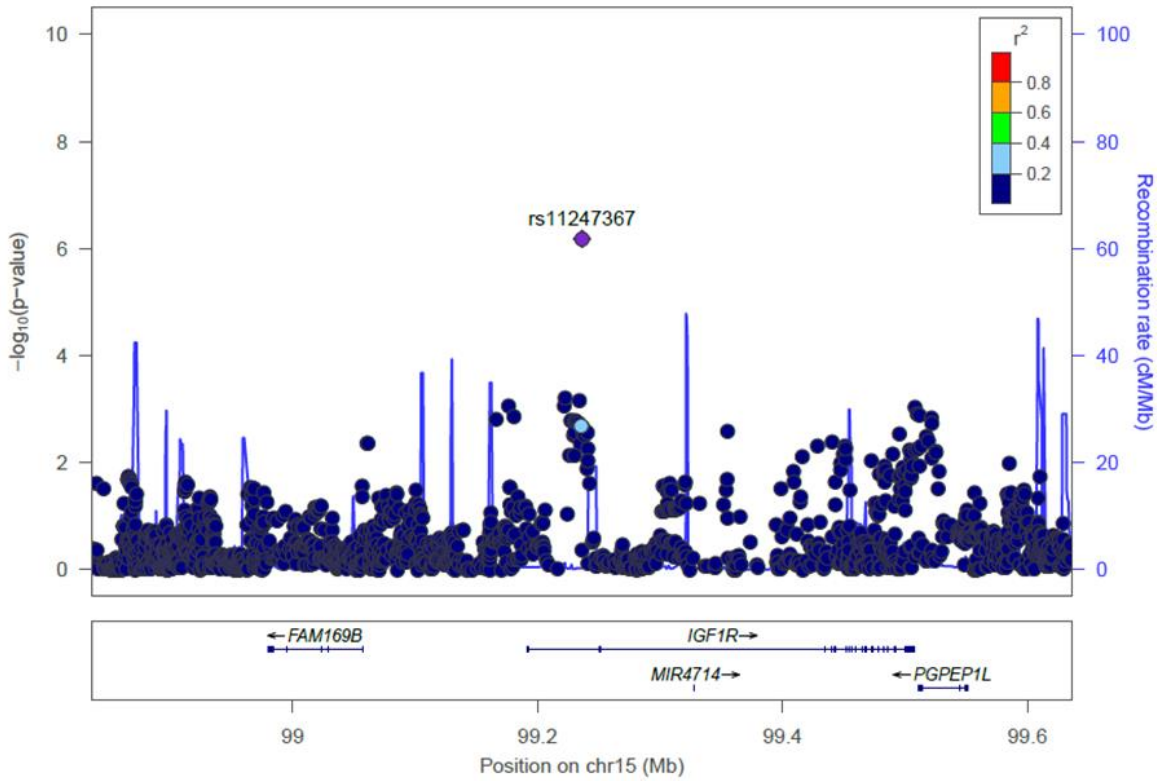

F

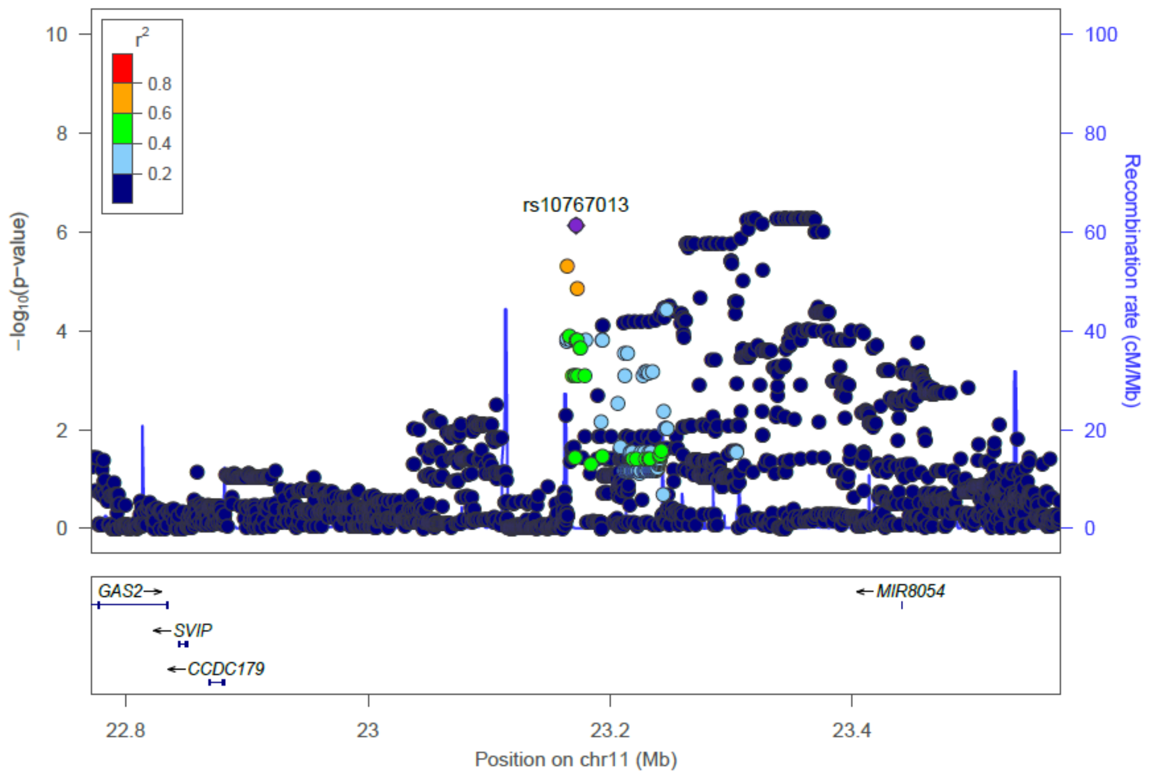

Supplementary Figure 3. Regional association plots for suggestively associated genetic variants: rs201666766 (A), rs170884 (B), rs4747268 (C), rs193145729 (D), rs11247367 (E) and rs10767013 (F).

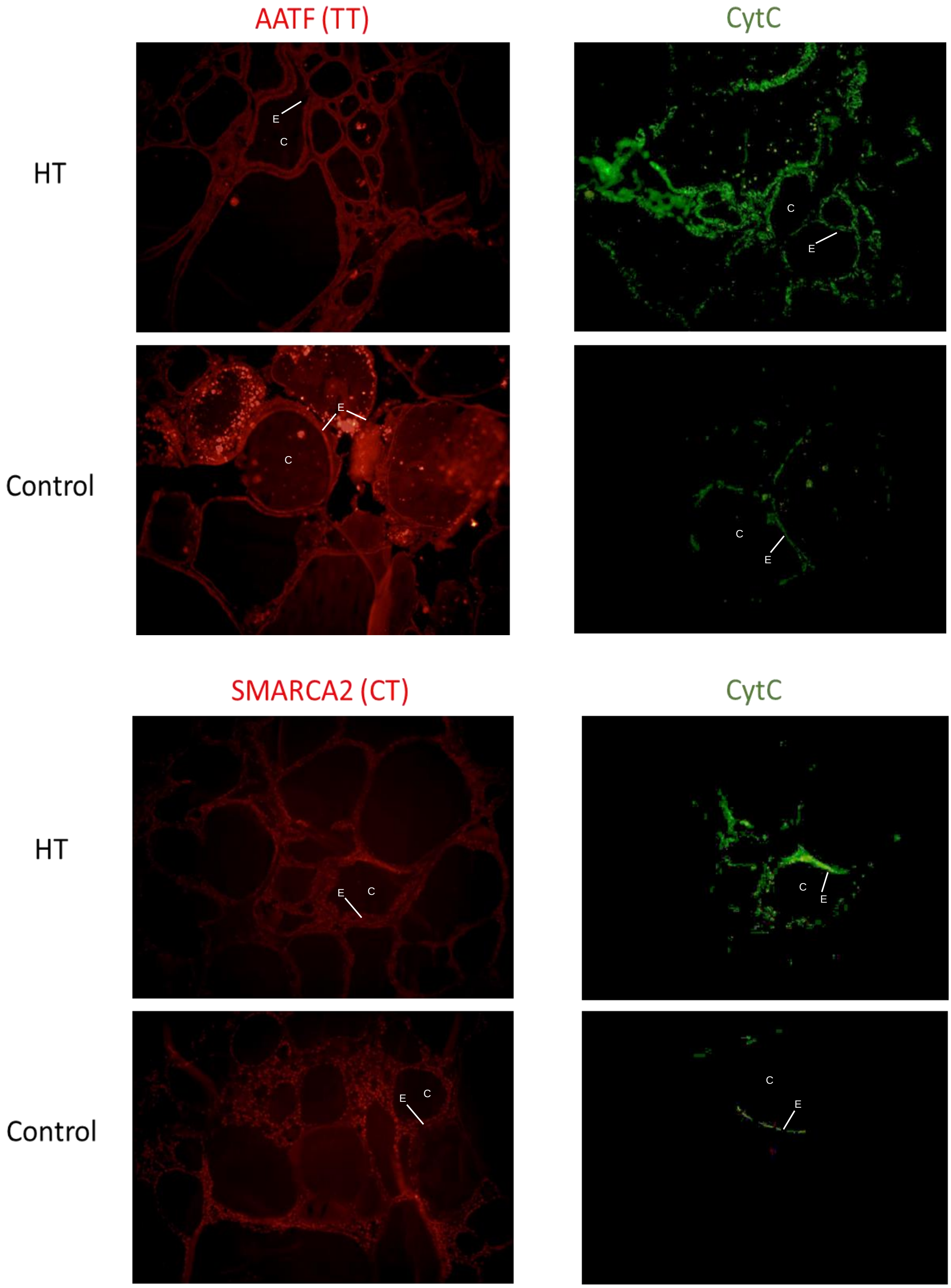

Supplementary Figure 4. Representative microphotographs of AATF and CytC expression in thyroid tissues of HT (n=3) and control (n=7) participants with rs7212416 TT genotype, and SMARCA2 and CytC expression in thyroid tissues of HT (n=5) and control (n=7) participants with rs10738556 CT genotype.

Microphotographs show spherical thyroid follicles containing colloid (C) lined with thyroid epithelia cells (E) called thyrocytes. Bright red signal represents positive staining for AATF and SMARCA2. AATF shows cytoplasmatic staining pattern in epithelial cells and extracellular staining pattern in colloid part of follicles. SMARCA2 displays nuclear staining pattern in thyroid epithelia cells. Green signal represents CytC expression which is located in cytoplasm of thyroid epithelia cells.
